# Characterization of the influence of cultivation parameters on extracellular modifications of antibodies during fermentation

**DOI:** 10.1186/1753-6561-7-S6-P85

**Published:** 2013-12-04

**Authors:** Christian Hakemeyer, Martin Pech, Gero Lipok, Alexander Herrmann

**Affiliations:** 1 Pharma Technical Development, Roche Diagnostics GmbH, Penzberg Germany

## Introduction

The production of protein-based medical agents, like monoclonal antibodies (Mabs), by biotechnological processes requires a comprehensive quality control. The pharmaceutical industry and national health authorities support the complete characterization of therapeutic proteins to increase the quality and safety. During numerous and different production steps like fermentation, purification and storage, various protein modifications on therapeutic products can occur, like deamidation of asparagine and glutamine, oxidation of methionine tryptophan residues, clipping of terminal amino acids, glycation and others.

During the development of fermentation processes, good growth conditions for the cell culture are of primary importance to obtain maximal productivity [1]. Until now only few efforts have been made to investigate the development of extracellular antibody modifications and their sources during fermentation as the first phase of the productions process. Already known is the fact that pH-value and temperature can induce modifications on monoclonal antibodies [2].

Aim of this work is to increase the knowledge about the development of extracellular modifications of monoclonal antibodies during the fermentation process. Therefore, parameters of fermentation were identified which influence modifications during cell-free incubation under common fermentation conditions (in shake flask and small scale bioreactor-systems).

## Results

The results from the shake flask experiments showed a different degree of changes of the charge isoform pattern (measured by IE-HPLC) for five analyzed antibodies during the approx. nine days of cell-free incubation. The respective increase of the amount of acidic regionwas strongly dependent on the specific protein. At the end of the incubation, the amount of the acidic region range from approx. 20 area-% to approx.75 area-% depending on the characteristics of the Mab. The increase in the acidic region correlated with a decrease of the main peak while the basic regionremained unchanged.

The specific influence of the parameters pH, temperature and dissolved oxygen (DO) on the modification of antibodies was further characterized in full factorial DoE designed experiments for three Mabs. For this purpose, cell broth was taken at an early stage from standard 1.000L fermentations with Chinese Hamster Ovary (CHO) cells and cells were removed by centrifugation. The cell-free supernatant was then transferred to small scale bioreactors and incubated for approx. ten days under the conditions listed in table 1.

**Table 1:** Setup for the small scale fermentation experiments

Experiment	pH	Temp. [°C]	DO [%]
1	6.7	33.0	45

2	6.7	40.0	5

3	7.0	36.5	25

4	7.0	36.5	25

5	7.3	33.0	5

6	7.3	40.0	45

7	6.7	40.0	45

8	6.7	33.0	5

9	7.0	36.5	25

10	7.0	36.5	25

11	7.3	33.0	45

12	7.3	40.0	5

In these experiments, elevated temperature conditions and higher pH values led to a faster modification (degradation) for all three investigated antibodies during the incubation compared to lower pH and temperature conditions, while dissolved oxygen level had no relevant impact on the kinetic of antibody degradation.

**Figure 1:**
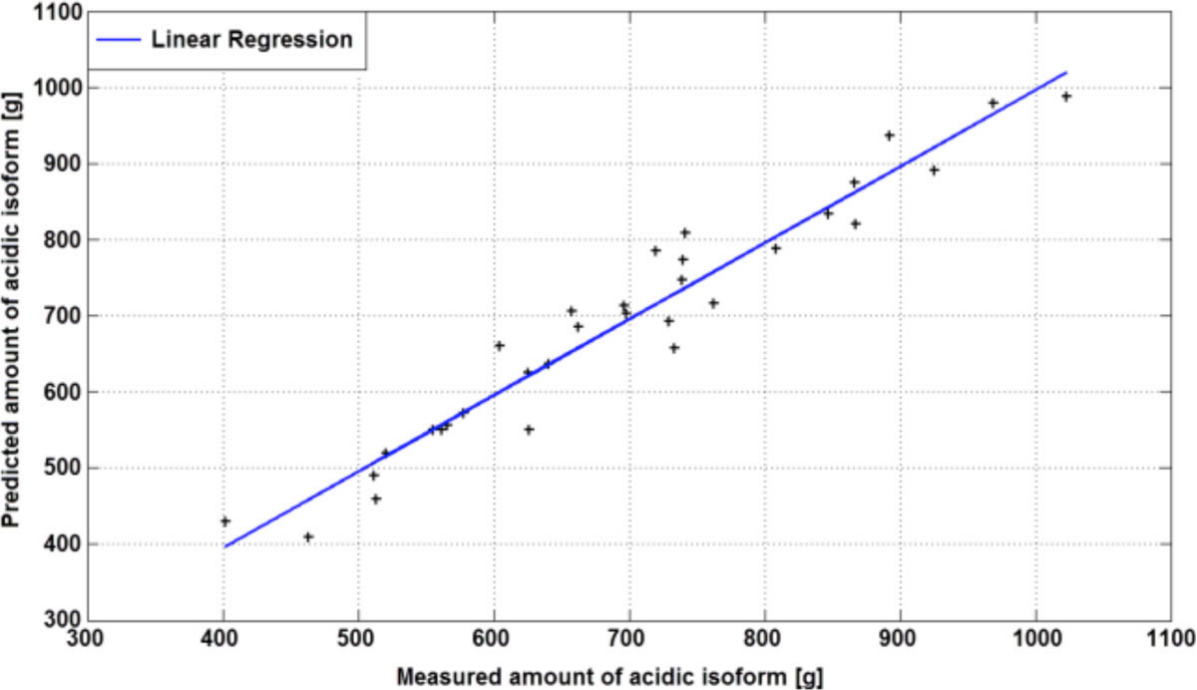
Correlation of measured versus calculated amount of acidic isoforms

The results of the cell-free incubation studies were used to develop a mathematical model was to predict the isoform pattern of the Mab during standard fermentations with CHO cells from inoculation to harvest. The amount of the acidic peak can be predicted, depending on the specific antibody characteristics as determined in the previous experiments, the concentration of the antibody during the cultivation, and the fermentation time and process conditions (pH, DO, temperature). Figure 1 shows an actual-by-predicted plot, comparing model predictions against measured values for several fermentations of one Mab. The model is well capable of predicting the amount of acidic isoform for this molecule.

## Conclusion

In this work, the influence of fermentation parameters (pH, DO, temperature) on the extracellular modification of Mabs (in the supernatant of cell broth) was examined. Higher temperature and higher pH values lead to a significant increase in the formation of the acid region species of Mabs compared to lower temperature and pH conditions. The impact of these process parameters on the modification kinetics of Mabs during cell-free incubation was characterized. Furthermore, additional modifications were detected, as oxidation, deamidation, generation of pyro glutamic acid, separation of lysin (data not shown).

The results of the incubation experiments in the small scale fermenter system lead to a mathematical prediction model for the increase of the acidic peak during a standard fermentation for the production of Mabs with CHO cells. This prediction model helps to develop robust fermentation processes.
